# X-ray velocimetry provides temporally and spatially-resolved biomarkers of lung ventilation in small airways disease

**DOI:** 10.1186/s12931-025-03295-6

**Published:** 2025-07-02

**Authors:** Bradley W. Richmond, Michael G. Lester, Vincent Lui, Jonathan Dusting, Sarath Raju, Gregory I. Snell, Jessica B. Blackburn, Katrina Douglas, Robert F. Miller, Trishul Siddharthan, Andreas Fouras

**Affiliations:** 1 Department of Veterans Affairs Medical Center, Nashville, TN USA; 2 Division of Allergy, Pulmonary, and Critical Care Medicine, Department of Medicine, Vanderbilt University Medical Center, Nashville, TN USA; 3 Department of Cell and Developmental Biology, Vanderbilt University, Nashville, TN USA; 4 4DMedical, 21255 Burbank Boulevard, Suite 120, Woodland Hills, CA 91367 USA; 5 Division of Pulmonary and Critical Care Medicine, Johns Hopkins University, Baltimore, MD USA; 6 Alfred Hospital, Melbourne, VIC Australia; 7 Division of Pulmonary, Allergy, and Sleep Medicine, Department of Medicine, University of Miami Miller School of Medicine, Miami, FL USA; 8 University of Melbourne, Melbourne, Australia

## Abstract

**Background:**

Small airways disease is a feature of many respiratory conditions. Currently available methods of diagnosing small airways lack sensitivity and/or cannot evaluate spatial heterogeneity. New diagnostic strategies for diagnosing small airways disease are needed.

**Methods:**

We determined the regional displacement of lung tissue calculated from fluoroscopic lung images acquired at multiple angles over sequential time points as a surrogate of ventilation. We applied this technique, which we call X-ray velocimetry (XV), to patients with chronic obstructive pulmonary disease (COPD) and impaired spirometry and veterans with deployment-related constrictive bronchiolitis (DR-CB) but preserved spirometry to determine XV-derived biomarkers specific for each condition.

**Results:**

We identified disease- and stage-specific XV biomarkers for COPD patients that correlated with airflow obstruction on spirometry. Further, we identified a set of XV-derived biomarkers that could distinguish veterans with DR-CB from controls despite normal spirometry in most patients from both groups.

**Conclusions:**

XV may provide a safe and widely-available strategy for diagnosing small airways disease while preserving spatial information. Future studies are required to validate the biomarkers described here in larger patient cohorts.

**Trial registration:**

Not required for this study. However, participants enrolled at VUMC were enrolled under ClinicalTrials.gov study NCT04489758 (submitted 07/23/2020).

**Supplementary Information:**

The online version contains supplementary material available at 10.1186/s12931-025-03295-6.

## Introduction

Spirometry is used to assess the volume of exhaled air over time and is the standard for lung function assessment [1]. Prolongation of expiratory time on spirometry is a marker of airflow obstruction and can result from luminal narrowing of the airways at any point along the bronchial tree as well as reduced recoil from loss of lung elasticity. However, due to their large number and high aggregate cross-sectional area, small (< 2 mm) airways contribute to a minority of airflow resistance and thus have little effect on exhalation time [2]. More than 50 years ago this idea led Mead to speculate small airways form a “quiet zone” of the lung where significant disease can accumulate without impacting spirometry [3]. Mead’s hypothesis has since been validated by pathologic studies showing significant narrowing or loss of small airways in lung tissue from patients with respiratory symptoms but no evidence of airflow obstruction on spirometry [4–6]. These subclinical alterations in small airway calibre have been linked to increased symptoms, respiratory-related disability, and other adverse outcomes [4, 7–9]. In addition to its insensitivity for small airways disease, spirometry provides a summative measure of pathology across all regions of the lungs, potentially masking important regional heterogeneity. Clinical computed tomography (CT) scanning could potentially show disease heterogeneity but small airways cannot current be visualized directly, although paired inspiratory-expiratory scans, combined with quantitative analysis, can assess for small airways disease indirectly [10].

X-ray velocimetry (XV) is a fluoroscopy-based technique that measures lung motion during tidal volume (resting) breathing as a four-dimensional (x, y, and z spatial dimensions plus time) surrogate of ventilation [11]. XV divides regions of the lung into voxels, tracks relative movement of features of the pulmonary parenchyma and associated structures in relation to one another, and measures lung expansion from the resulting displacement field. The application of XV in detecting lung pathology in vivo [12–17] has shown encouraging results. In addition, its correlation with established ventilation measurement in healthy individuals [11], as well as those with non-lung cancer malignancies [18], suggests a promising pathway for the use of XV in detecting lung pathology. Further exploration of XV in obstructive airway diseases could be beneficial to our understanding of these conditions and a potential role for XV in their diagnosis and monitoring. Here we assessed the discriminative accuracy of XV in patients with chronic obstructive pulmonary disease (COPD) and deployment-related constrictive bronchiolitis (DR-CB).

## Methods

### Study approval

The study protocol was approved by the Institutional Review Boards (IRBs) at The Alfred Hospital (IRB#274/21), Johns Hopkins University (IRB#00195043), the University of Miami Health System (IRB#20210065), and Vanderbilt University Medical Center (IRB#200473). Written informed consent was obtained prior to participation at each center. ClinicalTrials.gov registration was not required for this study. However, participants enrolled at VUMC were enrolled under ClinicalTrials.gov study NCT04489758 (submitted 07/23/2020).

### Participants

Participants were recruited from four academic medical centers in the United States and Australia (Supplemental Table 1). Across centers, inclusion criteria for control participants were: age > 18, no history of lung disease or current pulmonary symptoms, low-dose CT scan without evidence of chronic lung disease, and < 1 pack-year smoking history. Inclusion criteria for COPD participants included a diagnosis of COPD and FEV1/FVC ratio < 0.7. Inclusion criteria for DR-CB participants were: age > 18, prior military deployment to Iraq or Afghanistan, prior surgical lung biopsy demonstrating deployment-related constrictive bronchiolitis and prior CT scan of the chest.

### Pulmonary function tests (PFTs)

All participants underwent full PFTs including spirometry, lung volumes including total lung capacity (TLC), and diffusing capacity of the lungs for carbon monoxide (DLCO) within 6 months of XV.

### CT scans

To anatomically annotate fluoroscopy images, co-registration was performed with computed tomography (CT) scans of the chest. At VUMC, control participants underwent a low-dose screening CT of the lung. For veteran participants, high-resolution CT (HRCT) scans of the chest obtained for clinical purposes were used to minimize radiation exposure to study participants. The timing of CT scanning compared to XV does not significantly influence XV results.

### Fluoroscopy

X-ray velocimetry was performed using clinical fluoroscopes (Canon MEC XAC at The Alfred Hospital; Philips Azurion at Johns Hopkins University; Siemens Artis Zeego at the University of Miami Health System; Siemens Artis Zee at Vanderbilt University Medical Center). After supine positioning, participants were instructed to breathe normally. Cinefluoroscopic images were taken during one full respiratory cycle (end-exhalation through the beginning of the next exhalation) by directly visualizing lung excursion on fluoroscopy images in a separate room. Five sets of images were taken from different angles [direct anterior-posterior (AP), and four angles between + 72 and − 72 degrees relative to AP], integrated, and co-registered with CT scan images for anatomic annotation.

### X-ray velocimetry

X-ray velocimetry was performed using proprietary, fully automated XV Lung Ventilation Analysis Software (XV LVAS) from 4DMedical (Los Angeles, CA). XV LVAS reconstructs a spatiotemporal displacement field of the lung throughout the inspiratory phase of the breath from 2-dimensional (2D) measurements of lung motion captured from 5 different viewpoints/angles at a fixed acquisition rate (15 frames/second). Using X-ray fluoroscopy from these 5 different viewpoints, the inspiratory breath is divided into 7 equal phases of the breath, and XV LVAS reconstructs a 3-dimensional (3D) motion field over these 7 phases, resulting in a 4D spatially- and temporally-resolved displacement field. Different fields of interest are then computed from the displacement field as a surrogate of lung function. For this study, we derived three main fields of interest: (1) lung expansion which is computed via spatial differentiation of the displacement field; (2) the rate of lung expansion, computed as the inter-phase expansion per unit time; and (3) oscillation of the displacement and/or the flow field, which is defined as the change in direction of the field over the inspiratory phase of the breath. From these fields of interest, spatiotemporal biomarkers are computed via spatially- and temporally- aggregated statistics of these fields’ distributions. Examples of the spatiotemporally aggregated statistics that have been used in this study include: the average, inter-quartile range, entropy, skewness, kurtosis, and the percentage of the field having small or large values, among others.

There are 154 unique biomarkers used for the study where a biomarker is defined as a summary/point statistic over a field derived from the measurement of lung motion via X-ray velocimetry. Different combinations of these 154 unique biomarkers were explored to form composite biomarkers. Among the 154 individual biomarkers, 11 are derived from the local expansion field, 99 from the flow field, and 44 from the oscillation field. For all the fields considered in this study (expansion, airflow, oscillation), 11 spatially-resolved statistical quantities are computed from each field. For expansion, these are only computed at the final phase point (full inspiration). For flow, the 11 spatially-resolved statistics are calculated over 9 different time intervals to form 99 individual biomarkers. For oscillation, the field is already temporally resolved as the field is computed by considering all phase points in the breath. There are 4 different variants of oscillation that have been considered in this study (oscillation of the measured displacement filed, the derived flow field, and the square of each). As the study looked at n-dimensional composite biomarkers, these resulted in $\:\text{C}\left(\text{154,1}\right)+\text{C}\left(\text{154,2}\right)+\text{C}\left(\text{154,3}\right)+\text{C}\left(\text{154,4}\right)$ composite biomarkers being evaluated, where C(n, r) refers to the combination of a sample of size r from a dataset of size n. This yields 23,141,965 biomarkers.

### Statistical analysis

Binary classification was used to identify the most promising biomarkers derived from XV analysis. Logistic regression was performed to estimate a linear decision boundary that best separated the two classes (e.g. COPD or control). To evaluate the performance of a biomarker, the trade-off between sensitivity and specificity was quantified using the area under curve metric (AUC) of the receiver-operating-characteristic (ROC) plot. k-fold stratified cross-validation was performed to quantify the robustness of candidate biomarkers to perturbations in the data. Using k = 3, stratified cross-validation was repeated 100 times, where the order of the data is randomly perturbed each time. This produces multiple ROC curves, whose spread can be assessed for a candidate biomarker. Other statistical analyses (Student’s t-test and Pearson correlation coefficient) were computed using the Python open-source library SciPy version 1.9.1. Principal component analysis (PCA) was performed using R statistical software package version 4.4.1.

## Results

**Fig. 1:**
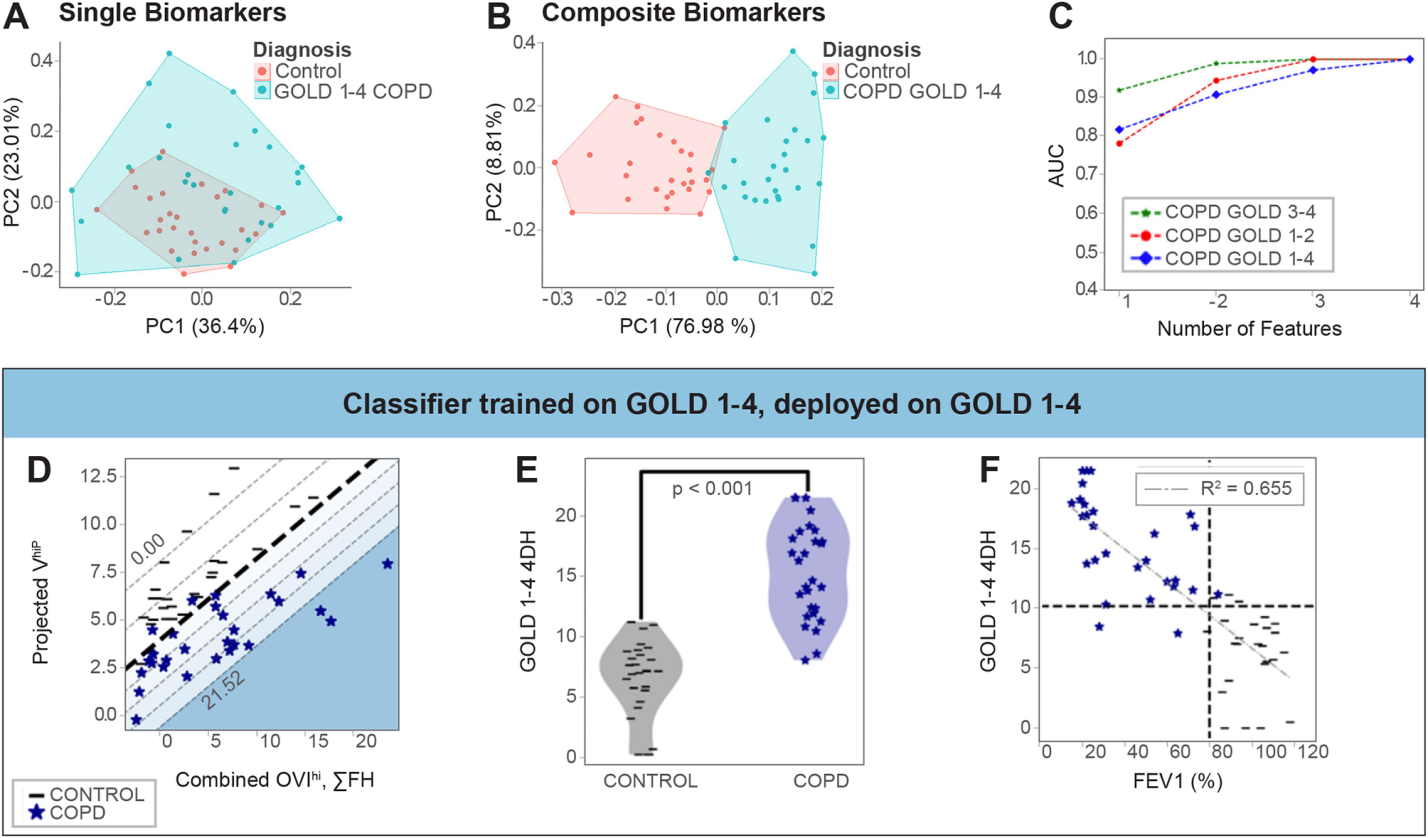
XV-derived biomarkers distinguish patients with COPD and obstructive spirometry from healthy controls. (**A**) Partial clustering is shown for the top 20 single biomarkers when plotted using the top two principal components on a principal component analysis (PCA) plot. (**B**) Near-complete separation is shown when repeating the PCA using composite biomarkers which combine both spatial and temporal information. (**C**) The AUC score improves as the number of dimensions increases from 1 to 3, and plateaus at a dimension of 4. (**D**) XV-derived biomarkers from GOLD 1–4 COPD patients presented as a 2D plot with axes representing the constituent features of the biomarkers and the colored contours representing four-dimensional heterogeneity (4DH) score. (**E**) 4DH score comparing GOLD 1–4 COPD patients to controls presented as violin plots. (**F**) 4DH score plotted against FEV1% predicted in GOLD 1–4 COPD patients. 4DH score and FEV1 are inversely correlated in COPD participants (*R*^2^ = 0.655, Pearson’s correlation coefficient). When considering only COPD participants, *R*^2^ = 0.308; when considering on control participants, *R*^2^ = 0.025. V^hiP^ = high ventilation volume percentage; OVI^hi^ = oscillation ventilation index high region; ∑FH = sum of flow heterogeneity

We first performed XV in participants with chronic obstructive pulmonary disease (COPD), a common obstructive lung disease characterized by small airways disease and emphysematous lung destruction [2, 19–21]. All COPD participants had spirometrically-confirmed disease (ratio of forced expiratory volume in the first second of exhalation to forced vital capacity, FEV1/FVC < 0.7) with a range of severities in airflow obstruction as defined by Global Initiative for Chronic Obstructive Lung Disease (GOLD) criteria [22]. Control participants were lifelong non-smokers with no known respiratory disease or symptoms and normal spirometry (Supplemental Table 1). We performed a principal component analysis (PCA) on the top 20 individual spatial or temporal biomarkers as ranked by their AUC. This was performed by decomposing an M-by-N matrix, where M represents the number of data points and N represents the feature. Every element in this M-by-N matrix represents the score for M^th^ datapoint given by the N^th^ biomarker. For both individual biomarkers and composite biomarkers, the value of this element represents the linear combination of the weight coefficients and the value of the biomarkers. We found poor segregation between GOLD 1–4 COPD participants and controls (Fig. 1A). In contrast, we analyzed the top 20 composite spatiotemporal biomarkers as ranked by their AUC and found near-complete segregation between GOLD 1–4 COPD participants and controls (Fig. 1B). Surprisingly, incorporating just 3 composite spatiotemporal biomarkers maximized AUC values (Fig. 1C, **blue line**): high ventilation volume percentage (V^hiP^), oscillation ventilation index high region (OVI^hi^), and sum of flow heterogeneity (∑FH). These biomarkers were combined to generate a four-dimensional ventilation heterogeneity (4DH) score which significantly differed between GOLD 1–4 COPD participants and controls and varied inversely with COPD disease severity as measured by FEV1 (*R*^2^ = 0.655, Pearson) (Fig. 1D-F). When COPD and control participants were considered independently, we observed a weak correlation between 4DH score and FEV1 in COPD participants (*R*^2^ = 0.308, Pearson) but not control participants (*R*^2^ = 0.025, Pearson), suggesting 4DH scores did not discriminate well between various levels of FEV1 in healthy individuals.

**Fig. 2:**
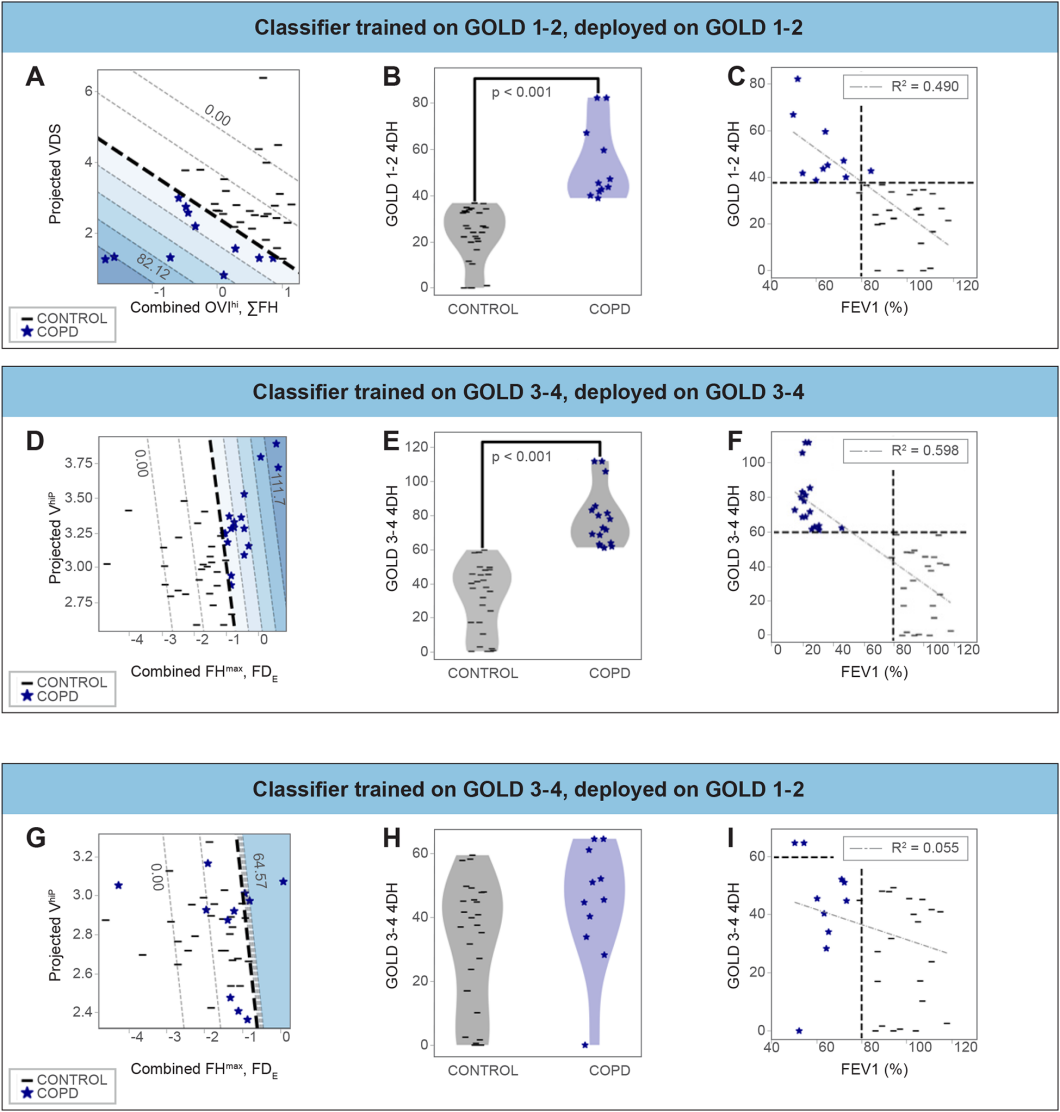
Stage-specific XV-derived biomarkers improve the diagnostic accuracy of XV relative to healthy controls. Each row shows XV biomarkers derived from COPD cohorts with different GOLD scores as indicated. For each cohort, the biomarkers are presented in 2D plots in the left column (**A**,** D**, and **G**) with axes representing the constituent features of the biomarkers and the colored contours representing four-dimensional heterogeneity (4DH) score. 4DH scores for each group of biomarkers is presented as violin plots in the middle column (**B**,** E**, and **H**). Statistical testing was performed using Student’s t-test and is indicated on the figure. The right column (**C**,** F**, and **I**) plots 4DH score against FEV1% predicted for each clinical group. V^hiP^ = high ventilation volume percentage; OVI^hi^ = oscillation ventilation index high region; ∑FH = sum of flow heterogeneity; VDS = ventilation distribution skewness; FH^max^ = maximum flow heterogeneity. FD_E_ = Shannon entropy of airflow distribution

Since cross-sectional imaging and pathologic studies indicate the pathology of COPD varies according to disease stage [10, 23–25] we speculated the most predictive XV-derived biomarkers might differ between early and advanced-stage disease. To assess this, we separately evaluated XV-derived biomarkers in participants with early-stage COPD (GOLD 1–2) and late-stage COPD (GOLD 3–4) relative to controls. As in the combined cohort, the value of additional spatiotemporal biomarkers plateaued after 3 in participants with GOLD 1–2 COPD (Fig. 1C, **red line**): ventilation distribution skewness (VDS), oscillation ventilation index (OVI^hi^) region, and sum of flow heterogeneity (∑FH). When these biomarkers were combined into a new 4DH score, we noted complete separation between GOLD 1–2 participants and controls (Fig. 2A-C). In contrast, while V^hiP^ remained the top predictive biomarker in GOLD 3–4 participants, maximum flow heterogeneity (FH^max^) and Shannon entropy of airflow distribution (FD_E_) emerged as the second and third-ranking biomarkers in participants with GOLD 3–4 COPD (Fig. 2D). Like GOLD 1–2 participants, these stage-specific spatiotemporal biomarkers completely segregated GOLD 3–4 participants from controls (Fig. 2D-F). However, when the GOLD 3-4-derived 4DH score was applied to participants with GOLD 1–2 disease, there was no difference in 4DH score between GOLD 1–2 and control participants (Fig. 2G-I) demonstrating FH^max^ and FD_E_ to be unique biomarkers for advanced COPD. Together, these results indicate XV can accurately distinguish between COPD cases and controls in this cohort and identify ventilation skewness as a unique biomarker in GOLD 1–2 COPD and FH^max^ and FD_E_ as unique biomarkers for GOLD 3–4 COPD.

**Fig. 3:**
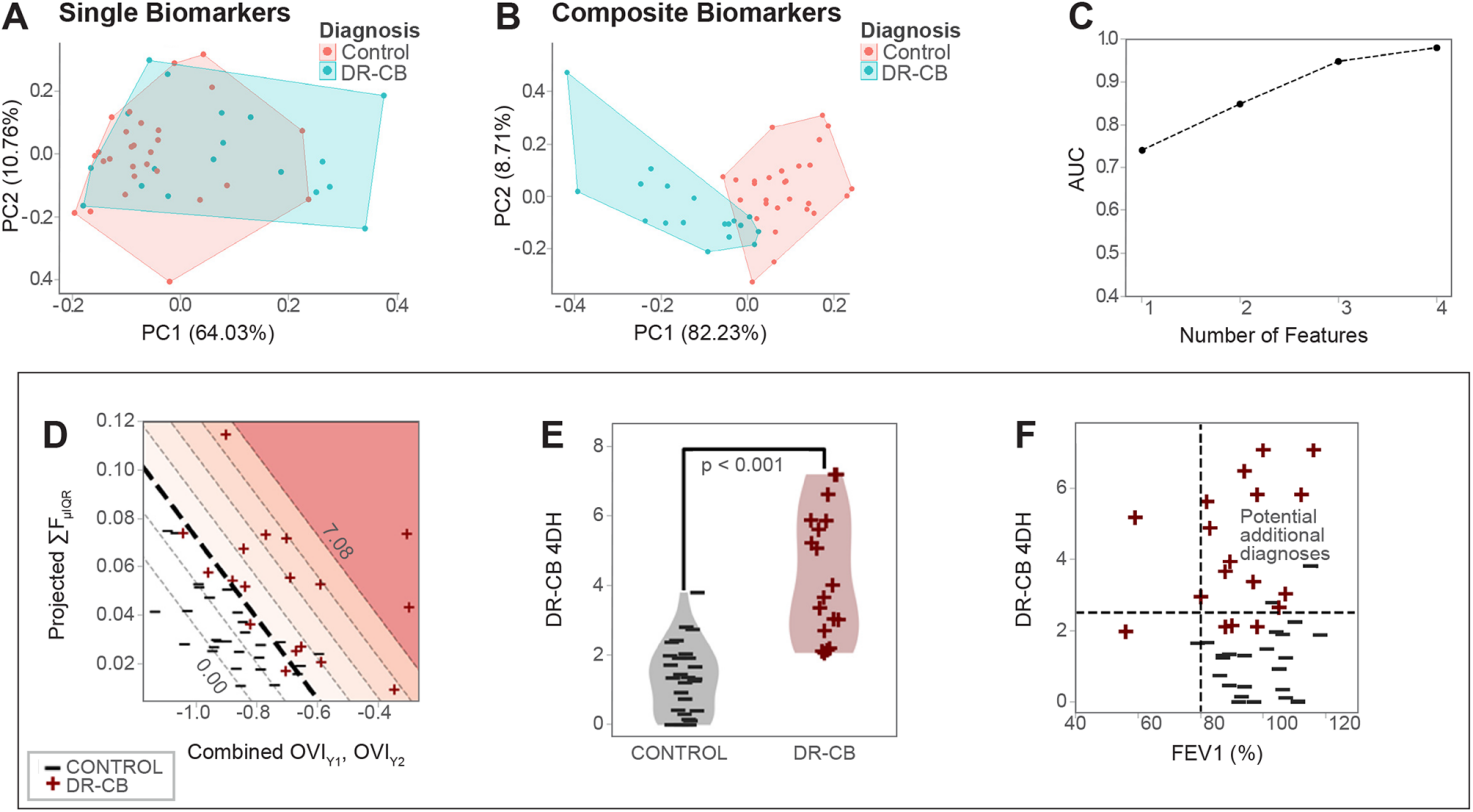
XV-derived biomarkers can distinguish patients with DR-CB and non-obstructive spirometry from healthy controls. (**A**) Similar to the COPD cohort, partial clustering is shown for the top 20 single biomarkers when plotted using the top two principal components as the basis. (**B**) Near complete separation is shown when repeating the PCA using composite biomarkers, again illustrating the importance of spatial and temporal information in helping to separate the two groups. (**C**) Similar to the COPD cohort, AUC improves as the number of dimensions increase from 1 to 3 and then plateauing thereafter. (**D**) XV biomarkers are presented in a 2D plot with axes representing the constituent features of the biomarker and the colored contours representing four-dimensional heterogeneity score (4DH). (**E**) 4DH score is presented as a violin plot. Student’s t-test was used to establish statistical significance as indicated on the figure. (**F**) Plot of 4DH score against FEV1% predicted in 18 DR-CB participants. Many DR-CB participants with normal FEV1% predicted (> 80%) have an abnormal 4DH score representing cases where 4DH score might be more sensitive than PFTs for detecting small airways disease. ∑F_µIQR_ = flow sum (mean x interquartile range); OVI_γ1_ = oscillation ventilation index skewness; OV_Iγ2_ = oscillation ventilation kurtosis

We next questioned whether XV could be used to identify small airways disease in individuals with preserved spirometry. Prior studies have shown that many Iraq and Afghanistan Veterans with otherwise unexplained dyspnea have pathologic evidence of constrictive bronchiolitis (CB, a type of small airways disease) on surgical lung biopsies despite having normal spirometry [4, 5]. We reasoned this cohort provided a unique opportunity to evaluate the potential for XV to detect small airways disease before it is apparent on spirometry. We recruited 18 veterans with surgical lung biopsy-proven, deployment-related constrictive bronchiolitis (DR-CB), 16 of which had no evidence of obstruction on PFTs (Supplemental Table 1). Similar to what we observed in COPD participants, composite spatiotemporal biomarkers better segregated DR-CB participants from controls compared to individual spatial or temporal biomarkers on PCA and the additive value of these composite biomarkers plateaued after 3 (Fig. 3A-C): flow sum mean x interquartile range (∑F_µIQR_), oscillation ventilation index skewness (OVI_γ1_), and OVI kurtosis (OVI_γ2_). The combined 4DH score generated from these biomarkers differed significantly between DR-CB and control participants (Fig. 3D-F). At a threshold 4DH score of 2.21, 13/16 (81%) of DR-CB participants with FEV1 > 80% predicted were classified as having an abnormal 4DH score (Fig. 3F). Of two patients with FEV1 < 80% predicted, one had an abnormal 4DH score and one had a normal score (Fig. 3F).

## Discussion

Small airways disease contributes to many respiratory conditions including asthma, COPD, constrictive bronchiolitis, and pulmonary complications after lung or hematopoetic stem cell transplantation but is challenging to diagnose in early stages. Here we leveraged an advanced fluoroscopic technique to derive biomarkers of small airways disease in COPD and DR-CB via XV. While our analysis utilized exhaustive, data-driven approaches to analyze millions of potential composite biomarkers from combinations of 154 individual biomarkers, we found that the value of this approach resides in a small number of biomarkers that integrate spatial and temporal information to distinguish participants with COPD or DR-CB from healthy controls, significantly reducing the complexity of future studies. We also note that these composite biomarkers likely reflect underlying pathophysiology of the studies diseases when assessed in multiple dimensions. In this way, XV may be better able to assess heterogenous disease than single-variable measurements such as densitometry. Although these results require validation in larger cohorts, they suggest XV is a promising strategy for regional assessment of specific pathologies known to affect the small airways disease that preserves spatial information and entails minimal cost and radiation exposure [11].

The pathology of COPD has been thoroughly described in cross-sectional radiogaphic and pathologic studies [2, 6, 10, 21, 23, 26, 27]. In early stages, COPD is characterized by small airway narrowing and dropout due to loss of elastin attachments tethering small airways to alveoli, fibrotic narrowing of the airway lumen, mucus plugging, and other factors [21, 28]. As the disease progresses, proteases produced by inflammatory cells in and around small airways cause degradation of the surrounding lung parenchyma leading to emphysema [21]. When comparing all COPD patients to healthy controls, we identified V^hiP^, OVI^hi^, and ∑FH as the top biomarkers of COPD. However, stage-specific analysis indicated that OVI^hi^ and ∑FH were more important in early-stage disease, where small airways disease predominates, while V^hiP^ was more important in late-stage disease, where small airways disease is accompanied by emphysema. Additionally, we found VDS is a unique biomarker for GOLD 1–2 COPD and FH^max^ and FD_E_ are unique biomarkers for GOLD 3–4 COPD. OVI^hi^, ∑FH, and VDS are all measures of flow heterogeneity over time, indicating discrepancy between flow patterns between timesteps, overall statistical heterogeneity of the calculated flow distribution, and higher-end clustering of greater flow values. It is important to note that ‘flow’ is imputed from regional spatial changes and threrefore may also reflect regional differences in compliance. We hypothesize that these biomarkers may arise from increased variability in airway luminal diameter, a relative increase in turbulent airflow, recruitment of mucus-plugged airways, and/or collateral airflow. In contrast, V^hiP^ may reflect areas of decreased elastic recoil and high compliance which occur in the setting of emphysema, reflecting increased local compliance as compared to regions of more intact parenchyma with greater recoil. We speculate that FH^max^ and FAD_E_, which were unique to advanced-stage COPD, may reflect a combination of these factors. Overall, the biomarkers in late-stage COPD might be associated with areas of decreased parenchymal recoil as well as an increased difference in compliance and flow rates between healthy and more affected regions of lung. A summary of these biomarkers, their definition, and their potential pathologic correlates is provided in Supplemental Table 2. Future studies, including well-characterized animal models of lung-disease and/or paired analysis of XV and pathology in human lungs ex vivo will ultimately be required to understand the relationship between XV-derived biomarkers and specific aspects of COPD pathology.

DR-CB is a pathologic finding that is difficult to diagnose non-invasively and emblematic of the poor sensitivity of existing methods of diagnosing small airways disease changes in general. We identified DR-CB biomarkers that were distinct from those identified in COPD and were able to classify many patients with biopsy-proven DR-CB and normal PFTs from healthy controls. Specifically, we identified increased ∑F_µIQR_, OVI_γ1_, and OVI_γ2_ as characteristic of DR-CB. These biomarkers correlate with disrupted smooth airflow, perhaps suggesting a trend toward increased turbulence. In addition, these biomarkers suggest there are a group of airways with increased overall airflow and temporal airflow variation and are consistent with variable areas of decreased airway distensibility and luminal narrowing, resulting in increased velocity distribution and areas of more highly variable flow (Supplemental Table 2). We find this to be a plausible finding based on the pathology of DR-CB, reflecting a more subtle form of change in airflow and/or tissue distensibility in specific areas that emerge as outliers in the flow distributons as compared to healthy controls. If these biomarkers are validated, XV could prove to be a non-invasive diagnostic option for veterans with DR-CB who otherwise could only be diagnosed with a surgical lung biopsy. Additionally, the success of XV for measuring small airways disease in the absence of spirometric obstruction suggests XV may be able to diagnose small airways disease after solid or hematopoetic stem cell transplantation or in pre-COPD, each of which represents a major unmet clinical need [29–31]. Studies comparing lung densitometry findings to the selected biomarkers might help elucidate the relative contribution to emphysema to the composite biomarkers in more advanced COPD with emphysema.

There are limitations of this study related to XV generally and our study specifically. Although the amount of radiation generated by a XV study is low and similar to that of a chest x-ray, anatomic registration currently requires a CT scan which exposes patients to additional radiation. However, a single CT scan (include a low-dose lung cancer screening CT scan) can be used for multiple XV studies and CT scans are often obtained as part of routine clinical care for COPD and DR-CB patients and in many other pulmonary diseases. Second, our study utilized clinical scanners which required co-localization of images taken from multiple angles during separate breaths. Future studies utilizing technology to obtain images during a single breath would improve data quality by eliminating breath-to-breath variability. Third, we cannot exclude the possibility that large airways disease in COPD patients or extrapulmonary factors such as ventilatory drive or diaphragmatic function in COPD or DR-CB contributed to some of the observed composite biomarkers. Fourth, our study did not include analysis of XV-derived biomarkers during forced exhalation as is done with PFTs. However, use of tidal volume breathing could reduce patient-level variability introduced during a forced expiratory maneuver and assessment of physiology during tidal volume breathing could generate new physiologic insights. Study-specific limitations include the lack of validation cohorts, with numerous combinations of biomarkers tested, which introduces the possibility of overfitting. Although this risk was mitigated through k-fold cross-validation [32] it will be critical to validate the biomarkers described here in additional patient cohorts.

## Conclusions

In summary, we used an agnostic approach to derive radiographic biomarkers of lung ventilation from the regional displacement of lung tissue on fluoroscopic images during resting breathing. Our results suggest XV may be leveraged to identify unqiue associated composite classification scores composed of newly assessed biomarkers.

## Electronic supplementary material

Below is the link to the electronic supplementary material.

Supplementary Material 1

## Data Availability

No datasets were generated or analysed during the current study.

## Abbreviations

4DH: 4 Dimensional heterogeneity
AP: Anterior-posterior
AUC: Area under the curve
COPD: Chronic obstructive pulmonary disease
CT: Computed tomography
DLCO: Diffusing capacity of the lung for carbon monoxide
DR-CB: Deployment-related constrictive bronchiolitis
FD_E_: Shannon entropy of airflow distribution
FEV1: Forced expiratory volume in the first second of exhalation
∑FH: Sum of flow heterogeneity
FH^max^: Maximal flow heterogeneity
FVC: Forced vital capacity
∑F_µIQR_: Flow sum (mean x interquartile range)
GOLD: Global initiative for chronic obstructive lung disease
HRCT: High-resolution computed tomography
IRB: Institutional review board
LVAS: Lung ventilation analysis software
OVI^hi^: Oscillation ventilation index high region
OVI_γ1_: Oscillation ventilation index skewness
OVI_γ2_: Oscillation ventilation index kurtosis
PCA: Principal component analysis
PFTs: Pulmonary function testing
ROC: Receiver operating curve
TLC: Total lung capacity
VDS: Ventilation distribution skewness
V^hiP^: High ventilation volume percentage
VUMC: Vanderbilt university medical center
XV: X-ray velocimetry
