# Supplementary material for: X-ray velocimetry provides temporally and spatially-resolved biomarkers of lung ventilation in small airways disease
Source: Respir Res. 2025 Jul 2;26:226. doi: 10.1186/s12931-025-03295-6 (PMC12224510; doi:10.1186/s12931-025-03295-6)
Supplement: Supplementary file 1 — Supplementary Material 1 [file 12931_2025_3295_MOESM1_ESM.docx]

**X-ray Velocimetry Provides Temporally and Spatially-Resolved Biomarkers of Lung Ventilation in Small Airways Disease**

**Supplemental Tables**

**Supplemental Table 1.** Patient demographics and clinical features. Values represent the overall cohort across centers.

|  | Age  (median, range) | Sex | Tobacco status | Pack-years  (median, range) | FEV1  (mean L, % pred) | FVC (mean L, % pred) | TLC  (mean L, % pred) | DLCO (% pred) |
| --- | --- | --- | --- | --- | --- | --- | --- | --- |
| Control (n=28)  Miami (n=22)  Vanderbilt  (n=6) | 42 (26-62) | 18M, 10F | All NS | N/A | 3.81 (101%) | 4.81 (103%) | 6.43 (99%) | 89% |
| COPD (n=26)  Alfred (n=13)  Johns Hopkins  (n=13) | 64 (52-79) | 15M, 11 F) | 20FS, 5 CS, 1 NS | 32 (0-98) | 1.15 (40%) | 2.79 (73%) | 7.46 (128%)  (of 13 available) | 44% (of 13 available) |
| DR-CB (n=18)  Miami (n=9)  Vanderbilt  (n=9) | 46 (41-62) | 13M, 5F | 12NS, 5FS, 1 ND | 1 (0-6) | 3.28 (91%) | 4.15 (90%) | 90% | 94% |

Abbreviations: COPD = chronic obstructive pulmonary disease; DR-CB = deployment-related constrictive bronchiolitis; M = male assigned at birth; F = female assigned at birth; N/A = not applicable; NS = never smoker, FS = former smoker; CS = current smoker; ND = no data available; FEV1 = forced expiratory volume in 1 second; FVC = forced vital capacity; TLC = total lung capacity; DLCO = diffusing capacity of the lung for carbon monoxide.

**Supplemental Table 2**. XV-derived markers, their definition, and potential pathologic correlates.

| **Biomarker** | **Abbreviation** | **Measurement** | **Disease correlate** | **Potential pathologic correlate** |
| --- | --- | --- | --- | --- |
| High ventilation volume percentage | V^hiP^ | Percentage of starting volumes of the lung with greater than 1.674 x the mean specific ventilation | All COPD, GOLD 3-4 COPD | Areas of decreased elastic recoil and relatively high compliance related to emphysematous changes |
| Oscillation ventilation index high region | OVI^hi^ | Variance in expansion of a specific volume of the lung across timesteps | All COPD, GOLD 1-2 COPD | Increased turbulent airflow, recruitment of mucous-plugged airways, colateral channel flow |
| Sum of flow heterogeneity | ∑FH | Overall summation of the variance of airflow distribution from the mean | All COPD, GOLD 1-2 COPD | Increased turbulent airflow, recruitment of mucous-plugged airways, colateral channel flow |
| Ventilation distribution skewness | VDS | Relative extremity in the distribution of volumetric change over time | GOLD 1-2 COPD | Changes in local elastic recoil with relative difference in regional compliance, variable airway resistance to flow |
| Maximum flow heterogeneity | FH^max^ | Maximal overall heterogeneity of airfow from the mean across timesteps | GOLD 3-4 COPD | Increased turbulent airflow, recruitment of mucous-plugged airways, colateral channel flow, variability in airway resistance and local elastic recoil |
| Shannon entropy of airflow distribution | FD_E_ | Maximum complexity and randomness of the airflow distribution | GOLD 3-4 COPD | Increased turbulent airflow, recruitment of mucous-plugged airways, colateral channel flow, variability in airway resistance and local elastic recoil |
| Flow sum (mean x interquartile range) | ∑F_µIQR_ | Overall summation of the variance of airflow multiplied by the mean | DR-CB | Diminished luminal cross-sectional area in affected airways resulting in greater velocity of airflow, increased work of breathing |
| Oscillation ventilation index skewness | OVI_γ1_ | Relative extremity in the distribution of the variance in expansion of the lung | DR-CB | Subpopulation of airways with decreased smoothness of airflow related to variable airway distensibility and luminal cross-sectional area, areas of increased turbulent flow |
| Oscillation ventilation index kurtosis | OVI_γ2_ | Relative tailedness in the distribution of the variance in expansion of the lung | DR-CB | Subpopulation of airways with decreased smoothness of airflow related to variable airway distensibility and luminal cross-sectional area, areas of increased turbulent flow |
